# Restriction-Spectrum Imaging of Bevacizumab-Related Necrosis in a Patient with GBM

**DOI:** 10.3389/fonc.2013.00258

**Published:** 2013-09-30

**Authors:** Nikdokht Farid, Daniela B. Almeida-Freitas, Nathan S. White, Carrie R. McDonald, Karra A. Muller, Scott R. VandenBerg, Santosh Kesari, Anders M. Dale

**Affiliations:** ^1^Department of Radiology, University of California San Diego, San Diego, CA, USA; ^2^Multimodal Imaging Laboratory, University of California San Diego, San Diego, CA, USA; ^3^Department of Radiology, University of São Paulo, São Paulo, Brazil; ^4^Department of Psychiatry, University of California San Diego, San Diego, CA, USA; ^5^Department of Pathology, University of California San Diego, San Diego, CA, USA; ^6^Department of Neurosciences, University of California San Diego, San Diego, CA, USA; ^7^Translational Neuro-Oncology Laboratories, Moores Cancer Center, University of California San Diego, San Diego, CA, USA

**Keywords:** restriction-spectrum imaging, diffusion-weighted imaging, bevacizumab, glioblastoma multiforme, necrosis

## Abstract

**Importance**: With the increasing use of antiangiogenic agents in the treatment of high-grade gliomas, we are becoming increasingly aware of distinctive imaging findings seen in a subset of patients treated with these agents. Of particular interest is the development of regions of marked and persistent restricted diffusion. We describe a case with histopathologic validation, confirming that this region of restricted diffusion represents necrosis and not viable tumor.

**Observations**: We present a case report of a 52-year-old man with GBM treated with temozolomide, radiation, and concurrent bevacizumab following gross total resection. The patient underwent sequential MRI’s which included restriction-spectrum imaging (RSI), an advanced diffusion-weighted imaging (DWI) technique, and MR perfusion. Following surgery, the patient developed an area of restricted diffusion on RSI which became larger and more confluent over the next several months. Marked signal intensity on RSI and very low cerebral blood volume (CBV) on MR perfusion led us to favor bevacizumab-related necrosis over recurrent tumor. Subsequent histopathologic evaluation confirmed coagulative necrosis.

**Conclusion and Relevance**: Our report increases the number of pathologically proven cases of bevacizumab-related necrosis in the literature from three to four. Furthermore, our case demonstrates this phenomenon on RSI, which has been shown to have good sensitivity to restricted diffusion.

## Introduction

We present a case of a 52-year-old man who first presented with a generalized tonic-clonic seizure. Subsequent MRI revealed a ring enhancing mass in the left frontal lobe. The patient underwent gross total resection, and pathology was consistent with GBM. Approximately 5 weeks later, the patient was started on standard combined chemotherapy and radiation (temozolomide at 75 mg/m^2^/day and involved-field radiation at a total dose of 60 Gy in 2.0 Gy fractions over a 6-week period). Concurrently, the patient was also started on bevacizumab as part of a clinical trial to assess the efficacy of bevacizumab for newly diagnosed GBM. Thereafter, the patient continued to receive adjuvant temozolomide and bevacizumab for 9 months.

**Figure 1:**
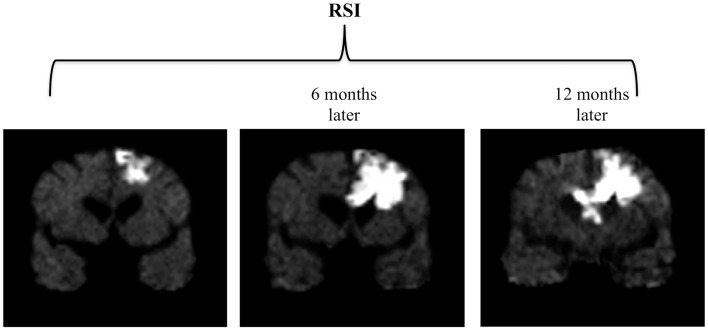
**Progression of findings on RSI over a 12-month period**. Three coronal RSI images spanning a 12-month period following surgery and chemoradiation depict an enlarging area of restricted diffusion which eventually crosses the corpus callosum and extends into the contralateral frontal lobe.

Sequential MRI’s were obtained during this time using an advanced diffusion-weighted technique called Restriction-Spectrum Imaging (RSI), which has been shown to provide improved conspicuity and delineation of high-grade primary and metastatic brain tumors compared with standard diffusion-weighted imaging (DWI) (1). Approximately 6 months following surgery, a small focal area of restricted diffusion appeared on the RSI sequence adjacent to the resection cavity. Over the next 18 months, this area of restricted diffusion became significantly larger and more confluent, eventually crossing the corpus callosum and extending into the contralateral frontal lobe (Figure 1). During this time, the patient also had progressive cognitive decline including increasing confusion, forgetfulness, and abulia. Therefore the imaging findings were initially interpreted as recurrent tumor and biopsy was considered.

**Figure 2:**
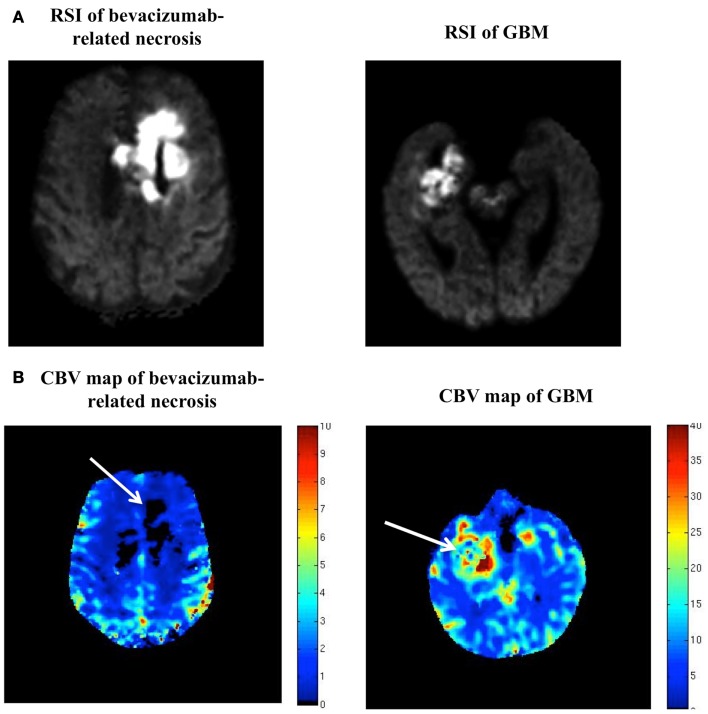
**(A,B)** RSI and CBV maps in bevacizumab-related necrosis versus GBM. **(A)** is a side-by-side comparison of the RSI signal seen in our patient and the RSI signal seen in a typical GBM, with the two RSI images scaled identically (i.e., same window and level). **(B)** is a side-by-side comparison of the DSC MR perfusion-generated CBV map for this patient and the CBV map for the same patient with GBM shown in **(A)** (with adjacent color scales).

However, on further inspection, it became clear that the degree and homogeneity of the restricted diffusion seen in this case was much greater than what is typically seen in high-grade glioma (Figure 2A), with GBM’s usually demonstrating less intense and more heterogeneous restricted diffusion. Furthermore, review of the patient’s dynamic susceptibility contrast (DSC) MR perfusion revealed that the cerebral blood volume (CBV) in the region of the restricted diffusion lesion was remarkably low – lower than that of the contralateral normal appearing white matter (NAWM). In contradistinction, high-grade gliomas typically demonstrate markedly elevated CBV (2, 3) (Figure 2B).

**Figure 3:**
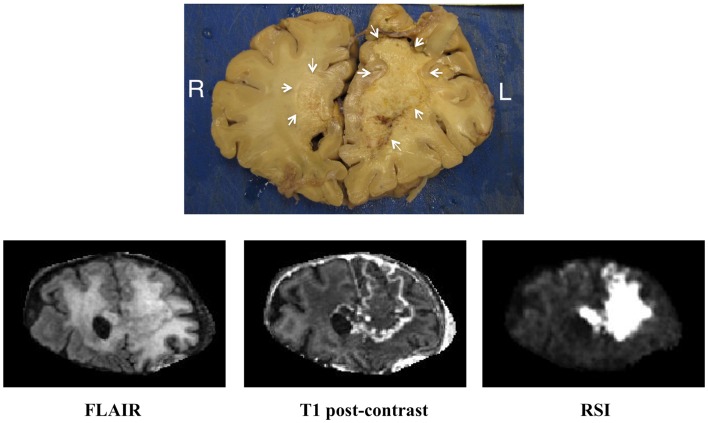
**Correlation of the gross pathology with imaging**. Coronal gross pathologic specimen as well as matched coronal FLAIR (fluid attenuated inversion recovery), coronal T1 post-contrast, and coronal RSI sequences from the patient’s last available MRI for correlation.

**Figure 4:**
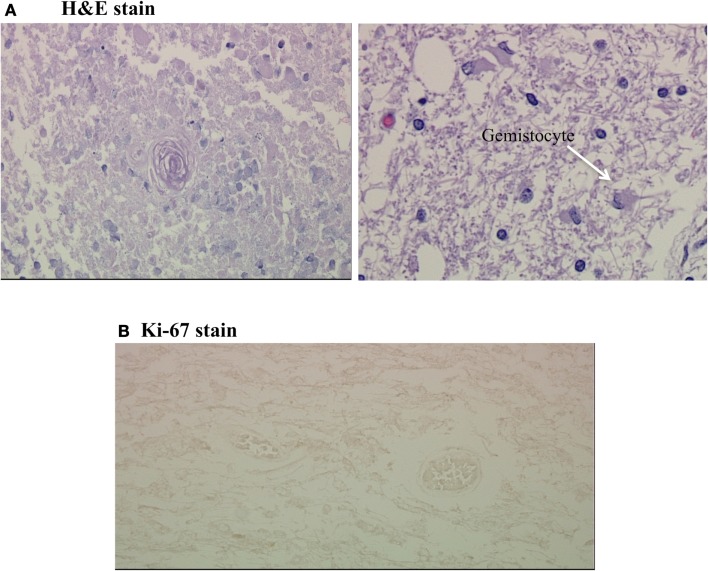
**(A,B)** Coagulative necrosis and negative Ki-67 stain. **(A)** Shows two H&E stains from the grossly necrotic region; the first demonstrates coagulative necrosis with a hyalinized fibrotic blood vessel in the center and the second demonstrates scattered gemistocytes on a background of edematous white matter. **(B)** is a Ki-67 stain of this region, which was completely negative.

Given the findings of marked signal intensity on the RSI sequence and very low CBV on DSC MR perfusion, it was concluded that this large region of restricted diffusion likely represented bevacizumab-related necrosis, despite the fact that it had enlarged over time and crossed the corpus callosum. Therefore, biopsy was not pursued, and the patient continued to be followed with sequential MRI’s. Approximately 2 years after initial diagnosis, the patient expired, and underwent autopsy. Gross pathologic evaluation demonstrated yellow, caseous material in the left frontal lobe compatible with necrosis (Figure 3). Histopathologic evaluation of this area revealed coagulative necrosis, gliosis, hyalinized blood vessels, and scattered atypical gemistocytes (Figure 4A). Although it is unclear if these gemistocytes represent tumor gemistocytes or reactive gemistocytic astrocytes, the Ki-67 stain was completely negative (Figure 4B), indicating that these are non-proliferating cells. In short, there was no evidence of proliferating, recurrent tumor at the time of autopsy.

## Background

Previous reports have described the development of regions of marked and persistent restricted diffusion in a subset of patients with malignant glioma during treatment with bevacizumab (4–6). Histopathologic data from these regions reveal necrosis and fibrotic, hyalinized blood vessels rather than viable tumor. Furthermore, these lesions have been correlated with improved outcomes (5). However, based on standard MR imaging, differentiating these lesions from areas of viable tumor, which are also associated with restricted diffusion (7, 8), remains challenging.

## Discussion

Although the development of regions of marked and persistent restricted diffusion has been described in a subset of malignant glioma patients treated with bevacizumab (4–6), this is still a fairly new phenomenon and has been pathologically proven in only three patients, with the present case increasing this number to four. Furthermore, these prior reports utilized standard DWI. In the present case, we describe this same phenomenon using a recently described advanced diffusion-weighted technique called RSI (9). In previous studies, RSI has shown greater sensitivity to restricted diffusion compared to standard DWI because it utilizes multiple *b*-values and diffusion times to separate out the spherically restricted water compartment from the hindered water compartment (9). As demonstrated in Figure 1, the degree and homogeneity of the RSI signal in this case is striking, facilitating the differentiation of this area of bevacizumab-related necrosis from recurrent tumor which demonstrates less intense and more heterogeneous restricted diffusion (Figure 2A).

Furthermore, we demonstrate that by utilizing DSC MR perfusion, we can further increase our diagnostic certainty in discriminating areas of bevacizumab-related necrosis from recurrent tumor. It is well known that MR perfusion of high-grade glioma yields elevated CBV (2, 3). In the present case, the area of bevacizumab-related necrosis demonstrated essentially no CBV, corroborating similar findings in prior reports (5, 6) and further differentiating this lesion from recurrent tumor.

The histopathologic findings in our case were very similar to those described in the prior reports including coagulative necrosis, gliosis, and hyalinized blood vessels (Figure 4A). However, our case also showed scattered atypical gemistocytes. Regardless of whether these gemistocytes represented tumor gemistocytes or reactive gemistocytic astrocytes, the Ki-67 stain was completely negative (Figure 4B), indicating that these cells were inactive non-proliferating cells and that no viable tumor was present.

Previous reports have proposed different explanations for the etiology of these areas of marked and persistent restricted diffusion. While one report has suggested that these lesions represent exacerbation of radiation necrosis by bevacizumab (4), others have suggested that these lesions result from bevacizumab-induced chronic hypoxia (6). We favor the latter explanation as it is well established that typical radiation necrosis actually demonstrates decreased signal on DWI, due to facilitation of diffusion related to liquefaction within the area of radiation necrosis (10). Further investigation is needed with larger patient cohorts and more histopathologic data, including immunohistochemistry, in order to determine the actual pathophysiology of these lesions.

## Concluding Remarks

In summary, we present a case of bevacizumab-related necrosis in a patient with GBM, emphasizing the distinctive appearance of this entity on RSI which, together with perfusion imaging, allowed us to differentiate this entity from recurrent tumor.

## Authors Contribution

Nikdokht Farid: author contributed to conception, acquisition, and interpretation of data. Author also participated in drafting the article. Daniela B. Almeida-Freitas: author contributed to conception, acquisition, and interpretation of data. Author also participated in drafting the article. Nathan S. White: author contributed in analysis and interpretation of data and participated in revising the article critically for important intellectual content. Carrie R. McDonald: author contributed to conception and interpretation of data. Author also participated in drafting the article. Karra A. Muller: author contributed in acquisition of data and participated in revising the article critically for important intellectual content. Scott R. VandenBerg: author contributed in acquisition of data and participated in revising the article critically for important intellectual content. Santosh Kesari: author contributed in acquisition of data and participated in revising the article critically for important intellectual content. Anders M. Dale: author contributed to conception and interpretation of data. Author also participated in revising the article critically for important intellectual content.

## Conflict of Interest Statement

The authors declare that the research was conducted in the absence of any commercial or financial relationships that could be construed as a potential conflict of interest.
